# Effect of Gender on Chronic Intermittent Hypoxic *Fosb* Expression in Cardiorespiratory-Related Brain Structures in Mice

**DOI:** 10.3389/fphys.2018.00788

**Published:** 2018-06-25

**Authors:** David M. Baum, Maud Saussereau, Florine Jeton, Carole Planes, Nicolas Voituron, Philippe Cardot, Marie-Noëlle Fiamma, Laurence Bodineau

**Affiliations:** ^1^Sorbonne Université, Institut National de la Santé et de la Recherche Médicale, UMR-S1158 Neurophysiologie Respiratoire Expérimentale et Clinique, Paris, France; ^2^Sorbonne Paris Cité, Université Paris 13, EA2363 Hypoxie et Poumon, Bobigny, France

**Keywords:** chronic intermittent hypoxia, FOSB/ΔFOSB, neuroplasticity, serotonin, sex hormones

## Abstract

We aimed to delineate sex-based differences in neuroplasticity that may be associated with previously reported sex-based differences in physiological alterations caused by repetitive succession of hypoxemia-reoxygenation encountered during obstructive sleep apnea (OSA). We examined long-term changes in the activity of brainstem and diencephalic cardiorespiratory neuronal populations induced by chronic intermittent hypoxia (CIH) in male and female mice by analyzing *Fosb* expression. Whereas the overall baseline and CIH-induced *Fosb* expression in females was higher than in males, possibly reflecting different neuroplastic dynamics, in contrast, structures responded to CIH by *Fosb* upregulation in males only. There was a sex-based difference at the level of the rostral ventrolateral reticular nucleus of the medulla, with an increase in the number of FOSB/ΔFOSB-positive cells induced by CIH in males but not females. This structure contains neurons that generate the sympathetic tone and which are involved in CIH-induced sustained hypertension during waking hours. We suggest that the sex-based difference in neuroplasticity of this structure contributes to the reported sex-based difference in CIH-induced hypertension. Moreover, we highlighted a sex-based dimorphic phenomenon in serotoninergic systems induced by CIH, with increased serotoninergic immunoreactivity in the hypoglossal nucleus and a decreased number of serotoninergic cells in the dorsal raphe nucleus in male but not female mice. We suggest that this dimorphism in the neuroplasticity of serotoninergic systems predisposes males to a greater alteration of neuronal control of the upper respiratory tract associated with the greater collapsibility of upper airways described in male OSA subjects.

## Introduction

Obstructive sleep apnea (OSA) is a breathing dysfunction characterized by collapse of the upper airways from atonia of upper airway and tongue muscles in the presence of continued diaphragmatic efforts during sleep (Dempsey et al., 2010). The origin of this collapse is multifactorial, with an anatomical predisposition to airway closure, such as adipose soft tissue deposition or compromised craniofacial structures and/or non-anatomical features, such as upper airway muscle responsiveness during sleep (Dempsey et al., 2010; Lévy et al., 2015). OSA leads to intermittent periods of hypoxemia/hypercapnia, followed by rapid return to normoxia caused by recurrent interruption or reduction in airflow (Almendros et al., 2010; Dewan et al., 2015), and is associated with cardiovascular and metabolic abnormalities (Somers et al., 1995; Lévy et al., 2015). In particular, an elevation of sympathetic nerve activity and sustained hypertension have been described as co-morbidities dependent on intermittent decreases in O_2_ (Fletcher et al., 1992b; Tamisier et al., 2009), because they are reduced by continuous positive airway pressure therapy during sleep among tolerant patients (Hedner et al., 1995; Dempsey et al., 2010).

The introduction of a model exposing conscious rats to repetitive episodes of hypoxia during the sleep period over several consecutive days, i.e., chronic intermittent hypoxia (CIH), by Fletcher et al. (1992b) has largely contributed to a better understanding of the physiological disorders induced by OSA. Rats subjected to CIH develop hypertension dependent on an increase in peripheral chemoreceptor activity and the activity of the sympathetic nervous system, the renin-angiotensin system, and endothelin (Lesske et al., 1997; Fletcher et al., 2002; Prabhakar et al., 2007; Dempsey et al., 2010; Capone et al., 2012; Lévy et al., 2015). Alteration of the baseline central respiratory drive and its adaptation to hypoxia has also been reported (Baker and Mitchell, 2000; Morgan et al., 2016). Some of the mechanisms involved in these physiological alterations have been more recently delineated. The repetitive succession of hypoxemia-reoxygenation, which causes oxidative stress because of the accumulation of reactive oxygen species (ROS), is involved in potentiation of the hypoxic chemosensory response of peripheral chemoreceptors (Iturriaga et al., 2009; Del Rio et al., 2010). The use of the long-term activity marker FOSB/ΔFOSB as a neuroplasticity marker (Malik et al., 2005) has highlighted sustained modulations in cardiorespiratory-related brain structures by CIH (Knight et al., 2011, 2013; Cunningham et al., 2012; Bathina et al., 2013; Saxena et al., 2015; Faulk et al., 2017). Also, CIH has been reported to alter the density of serotonin (5-HT) and noradrenaline terminals in the hypoglossal nucleus (12N), which contains motoneurons that innervate upper airway and tongue muscles (Rukhadze et al., 2010), suggesting that CIH *per se* contributes to upper airway instability, since upper airway muscle tonus is dependent on 5-HT signaling, especially during sleep (Jelev et al., 2001; Sood et al., 2003).

Although OSA is clearly a sex-dependent disease, the previously reported mechanistic processes have mainly been revealed by studies performed only on males. OSA has been shown to be two to three times more prevalent in men than women in several general population studies (Young et al., 1993; Bixler et al., 2001; Peppard et al., 2013) and is three to four times higher in menopausal than pre-menopausal women (Bixler et al., 2001; Young et al., 2003). A plausible hypothesis is that progesterone contributes to limit the occurrence of OSA; progesterone is a well-known potent respiratory stimulant (Joseph et al., 2013) and a correlation between low progesterone levels and high OSA frequency has been reported in pregnant women (Lee et al., 2017). In addition, a lower prevalence of hypertension in women than men diagnosed with OSA has been reported (Huang et al., 2008; Yu et al., 2014), although another study concluded that there was no sexual dimorphism in the prevalence of hypertension (Hermans et al., 2014). Such a sex-dependent difference in cardiovascular physiological disorders induced by OSA has been supported by CIH studies in rats, showing that males develop more severe hypertension than females (Hinojosa-Laborde and Mifflin, 2005). The difference in the consequence of OSA/CIH between males and females could be reasonably due to the antioxidant properties of estradiol (Moorthy et al., 2005b; Arevalo et al., 2015), which prevents the cardiorespiratory disorders and oxidative stress induced by CIH (Laouafa et al., 2017).

Here, we examined CIH-induced changes in the activity of brainstem and diencephalic cardiorespiratory neuronal populations in male and female mice to characterize the impact of sex on the neuroplasticity encountered in OSA. Our working hypothesis was that the neuroplasticity to CIH was different depending on sex, and thus differs between males and females. We assessed the neuroplasticity to CIH by immunodetection of the long-term neuronal markers FOSB/ΔFOSB. We chose FOSB/ΔFOSB detection rather than classically used c-FOS detection because the latter does not reflect intermittent stimulation of neurons, such as that induced by CIH, because of the rapid degradation of c-FOS (Knight et al., 2011). Dual labeling allowed us to investigate the catecholaminergic, serotoninergic, and orexinergic character of the FOSB/ΔFOSB-positive cells; these neuronal populations have all been shown to be involved in cardiorespiratory adaptation.

## Materials and methods

### Animals and CIH induction

Experiments were performed on both male and female 10-week-old C57BL/6JRj mice (Janvier Laboratories, France).

All experiments were approved by the Charles Darwin Ethics Committee for Animal Experimentation (APAFIS#1258) and were carried out in accordance with Directive 2010/63/EU of the European Parliament and the Council of 22 September 2010 and French law (2013/118). All efforts were made to minimize the number of animals used and their suffering. Animals were maintained on a 12-h light-dark cycle with free access to food and water.

Prior to CIH exposure, animals were allowed to acclimate for 1 week to the experimental chamber. Then, 12 and 11 randomly selected male and female mice (males 24.95 ± 0.62 g; females 19.06 ± 0.47 g) were subjected to daily sine-like computer-assisted O_2_ oscillations of 40 cycles per hour from 9:00 a.m. to 5:00 p.m. (animals' sleep period) for 21 consecutive days (O_2_ Sense Gas Driver Vivo, Adelbio, Aubière, France). In each cycle, O_2_ was reduced from 21 to 6% FiO_2_ over 35 s (inhaled dioxygen fraction) by injection of N_2_ into the chamber, followed by 55-s flush with compressed air, until the FiO_2_ again reached 21%. This protocol resulted in an arterial oxyhemoglobin saturation nadir of approximately 60% in the mice, as for similar previously described protocols (Reinke et al., 2011; Chodzynski et al., 2013; Schulz et al., 2014; Gille et al., 2018). In parallel, 12 male and 11 female sham mice (males 25.87 ± 0.36 g; females 19.69 ± 0.48 g) were subjected to identically-timed air exchanges but with the FiO_2_ maintained at 21%. O_2_ levels were continuously measured in the chambers during both the CIH and sham procedures using an O_2_ detector (Oxygen sensor KE-25F3, GS Yuasa, CE).

### Brain insolation and sectioning

After the 21st day of CIH or sham treatment, mice were deeply anesthetized with an intraperitoneal injection of pentobarbital (Nembutal®; 60 mg/kg) and prepared for FOSB/ΔFOSB immunochemistry. The genital areas of female mice were photographed for a later determination of the phase of the ovarian cycle, as published (Byers et al., 2012). Mice were transcardially perfused with 0.9% saline-buffered solution, followed by 4% paraformaldehyde in 0.1 M phosphate buffer (PB; pH 7.4). After fixation, brains were removed and post-fixed by immersion in the same fixative solution for 36 h at 4°C. They were then dehydrated for 4 days by immersion in a 0.1 M PB solution containing 30% sucrose at 4°C. The neuraxis was coronally cut from the caudal edge of the medulla oblongata to the rostral edge of the hypothalamus into three sets of serial 40-μm-thick sections with a cryostat (Leica CM 1510S). Sections were collected in 0.1 M PB solution containing 30% sucrose, 30% ethylene glycol, 1% polyvinylpyrrolidone, and 0.5%NaCl and stored at −20°C until immunohistochemical staining.

### Immunohistochemistry

#### FOSB/ΔFOSB immunohistochemistry

Every third section was processed for FOSB/ΔFOSB-like immunohistochemistry, using procedures based on previously published work (Voituron et al., 2006; Bodineau et al., 2011; Perrin-Terrin et al., 2016). Sets of sections from CIH and control mice were processed in parallel. Sections were incubated with a rabbit polyclonal antibody against FOSB/ΔFOSB (sc-7203; Santa Cruz Biotechnology Inc., Santa Cruz, CA, USA; 1:500) for 48 h at 4°C. They were then incubated for 2 h with a biotinylated goat anti-rabbit immunoglobulin (Vector Laboratories, Burlington, Canada; 1:500), followed by an avidin-biotin-peroxidase complex (ABC; PK-6100; Vector Laboratories; 1:250) for 1 h. Peroxidase activity was detected using 0.015% 3,3′-diaminobenzidine tetrahydrochloride (04001; Biovalley), 0.4% Nickel ammonium sulfate hexahydrate (464545; Carlo Erba reagent), and 0.006% H_2_O_2_ (H1009; Sigma) in 0.05M Tris-HCl buffer (pH 7.6).

Control sections were processed in parallel, but with the omission of either primary or secondary antibodies; no labeling was observed under such conditions.

Sections were then washed, mounted in a sequential caudo-rostral order on silane-treated slides, dehydrated with absolute ethanol (purity ≥ 99.5%; Merck, 107017), cleared with xylene, and coverslipped using Entellan® (Merck, 107960).

#### Coupling of the FOSB/ΔFOSB immunohistochemistry with tyrosine hydroxylase, 5-HT, and orexin

The detection of FOSB/ΔFOSB was coupled with that of tyrosine hydroxylase (TH), 5-HT, and orexin to characterize the FOSB/ΔFOSB-positive cells in series of retained cuts. Dual detection of FOSB/ΔFOSB and TH and FOSB/ΔFOSB and 5-TH was performed on brainstem sections and that of FOSB/ΔFOSB and orexin on sections from the diencephalon. FOSB/ΔFOSB was first detected according to the same protocol as above. The free-floating sections were then incubated with either a mouse monoclonal anti-TH antibody (MAB318, Millipore, 1:4,000), a rabbit polyclonal anti-5-HT antibody (S5545, Sigma–Aldrich, Saint-Quentin Fallavier, France; 1:500; 48 h; 4°C), or a goat polyclonal anti-orexin A antibody (Santa Cruz, sc-8070; 1:6,000; 48 h; 4°C). Sections were subsequently incubated for 2 h with adapted biotinylated antibodies: i.e., horse anti-mouse (Vector Laboratories, Burlington, Canada; 1:500), goat anti-rabbit (Vector Laboratories, Burlington, Canada; 1:500), or horse anti-goat (Vector Laboratories, Burlington, Canada; 1:500) antibodies. They were then incubated with ABC (1:250). The TH, 5-HT, and orexin immunoreactivities were detected by incubation for 3 to 5 min with 0.015% 3,3′-diaminobenzidine tetrahydrochloride and 0.006% H_2_O_2_ in 0.05 M Tris-HCl buffer (pH 7.6).

For all dual detections, the control sections were processed in parallel, but with the omission of either primary or secondary antibodies; no labeling was observed under such conditions.

Sections were then washed, mounted in sequential caudo-rostral order on silane-treated slides, dehydrated with absolute alcohol, cleared with xylene, and coverslipped using Entellan® (Merck, 107960).

### Quantitative analysis of the effect of chronic intermittent hypoxia on the number of FOSB/ΔFOSB-positive cells and their characterization

**Figure 1:**
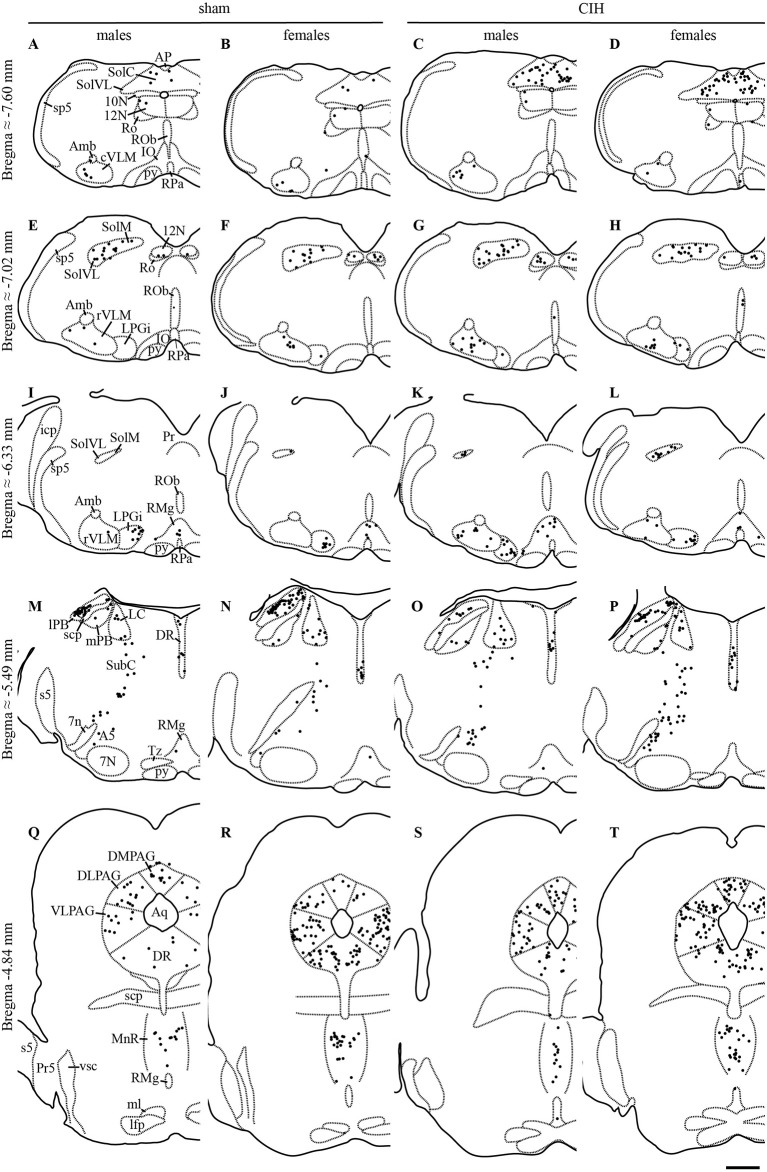
FOSB/ΔFOSB-positive cells in cardiorespiratory-related structures in the brainstem. Drawings of representative sections from the medulla oblongata **(A–L)**, pons **(M–P)**, and mesencephalon **(Q–T)**, illustrating the distribution of FOSB/ΔFOSB-positive cells (black dots) in cardiorespiratory-related brainstem structures under sham conditions **(A,B,E,F,I,J,M,N,Q,R)** and CIH **(C,D,G,H,K,L,O,P,S,T)** in male **(A,C,E,G,I,K,M,O,Q,S)** and female **(B,D,F,H,J,L,N,P,R,T)** mice. Scale bar for all drawings = 500 μm. 7n, facial nerve; 7N, facial nucleus; 10N, dorsal motor nucleus of vagus; 12N, hypoglossal nucleus; A5, A5 region; Amb, ambiguus nucleus; AP, area postrema; Aq, aqueduct; cVLM, caudal part of the ventrolateral reticular nucleus of the medulla; DLPAG, dorsolateral periaqueductal gray; DMPAG, dorsomedial periaqueductal gray; DR, dorsal raphe nucleus; icp, inferior cerebellar peduncle; IO, inferior olive; LC, locus coeruleus; lfp, longitudinal fasciculus of the pons; lPB, lateral parabrachial nucleus; LPGi, lateral paragigantocellular nucleus; ml, medial lemniscus; MnR, median raphe nucleus; mPB, medial parabrachial nucleus; Pr, prepositus nucleus; Pr5, principal sensory trigeminal nucleus; py, pyramidal tract; RMg, raphe magnus nucleus; Ro, nucleus of Roller; ROb, raphe obscurus nucleus; RPa, raphe pallidus nucleus; rVLM, rostral part of the ventrolateral reticular nucleus of the medulla; s5, sensory root of the trigeminal nerve; scp, superior cerebellar peduncle; SolC, nucleus of the solitary tract, commissural part; SolM, nucleus of the solitary tract, medial part; SolVL, nucleus of the solitary tract, ventrolateral part; sp5, spinal trigeminal tract; SubC, subcoeruleus nucleus; Tz, nucleus of the trapezoid body; VLPAG, ventrolateral periaqueductal gray; vsc, ventral spinocerebellar tract.

**Figure 2:**
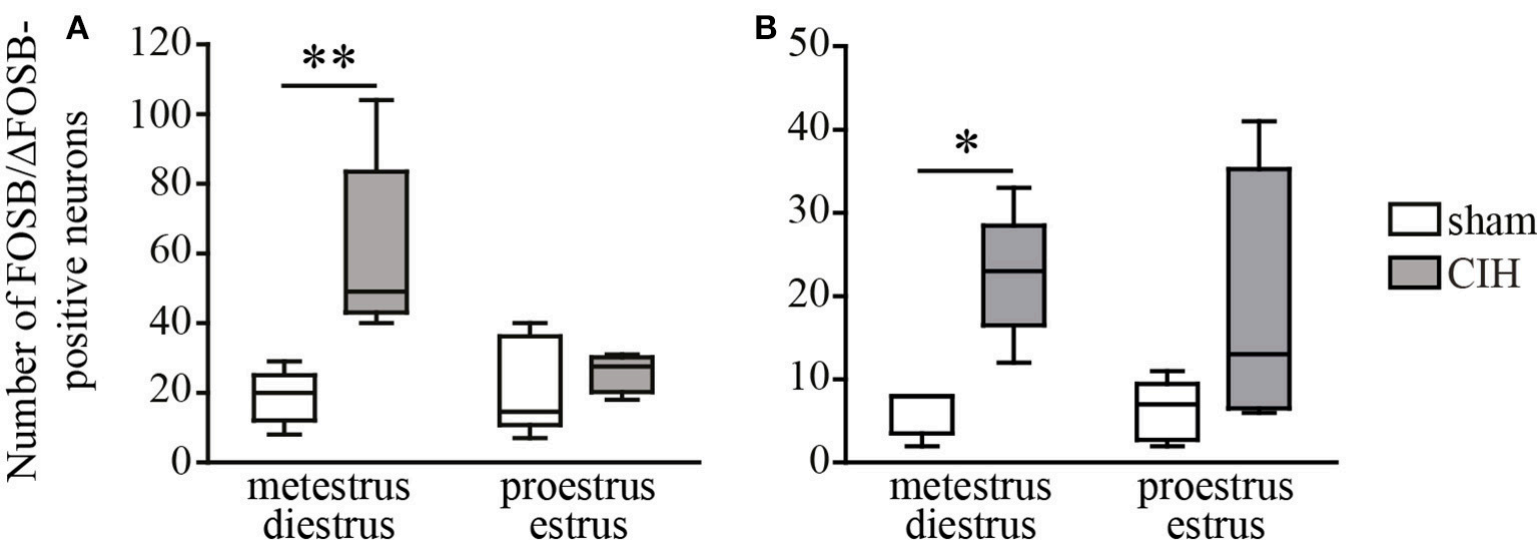
CIH-induced changes in the number of FOSB/ΔFOSB-positive cells in the SolC and the A5 depending on the phase of the ovarian cycle. Box plot of the number of FOSB/ΔFOSB-positive cells in the SolC **(A)** and A5 **(B)**, showing the median, quartiles, minima, and maxima during metestrus/diestrus and proestrus/estrus in female mice subjected to CIH or sham conditions. Sham, *n* = 5 for metestrus/diestrus and *n* = 6 for proestrus/estrus; CIH, *n* = 5 for metestrus/diestrus and *n* = 4 for proestrus/estrus. ^*^p < 0.05, ^**^p < 0.01.

**Figure 3:**
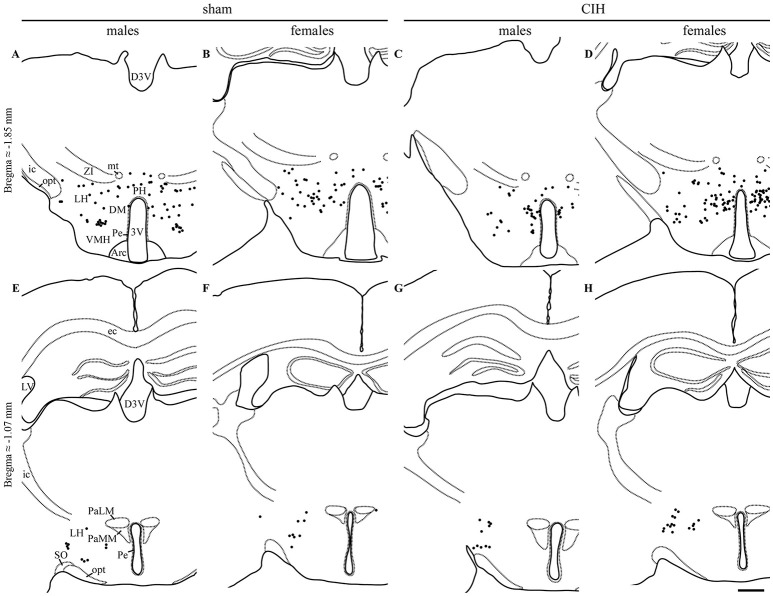
FOSB/ΔFOSB-positive cells in cardiorespiratory-related structures in the diencephalon. Drawings of representative sections from the caudal **(A–D)** and rostral diencephalon **(E–H)**, illustrating the distribution of FOSB/ΔFOSB-positive cells (black dots) in cardiorespiratory-related structures under sham conditions **(A,B,E,F)** and CIH **(C,D,G,H)** in male **(A,C,E,G)** and female **(B,D,F,H)** mice. Scale bar for all drawings = 500 μm. 3V, 3rd ventricle; Arc, arcuate hypothalamic nucleus; D3V, dorsal 3rd ventricle; DM, dorsomedial hypothalamic nucleus; ec, external capsule; ic, internal capsule; icp, inferior cerebellar peduncle; LH, lateral hypothalamic area; LV, lateral ventricle; mt, mammillothalamic tract; opt, optic tract; PaLM, paraventricular hypothalamic nucleus, lateral magnocellular part; PaMM, paraventricular hypothalamic nucleus, medial magnocellular part; Pe, periventricular hypothalamic nucleus; PH, posterior hypothalamic area; SO, supraoptic nucleus; VMH, ventromedial hypothalamic nucleus; ZI, zona incerta.

**Figure 4:**
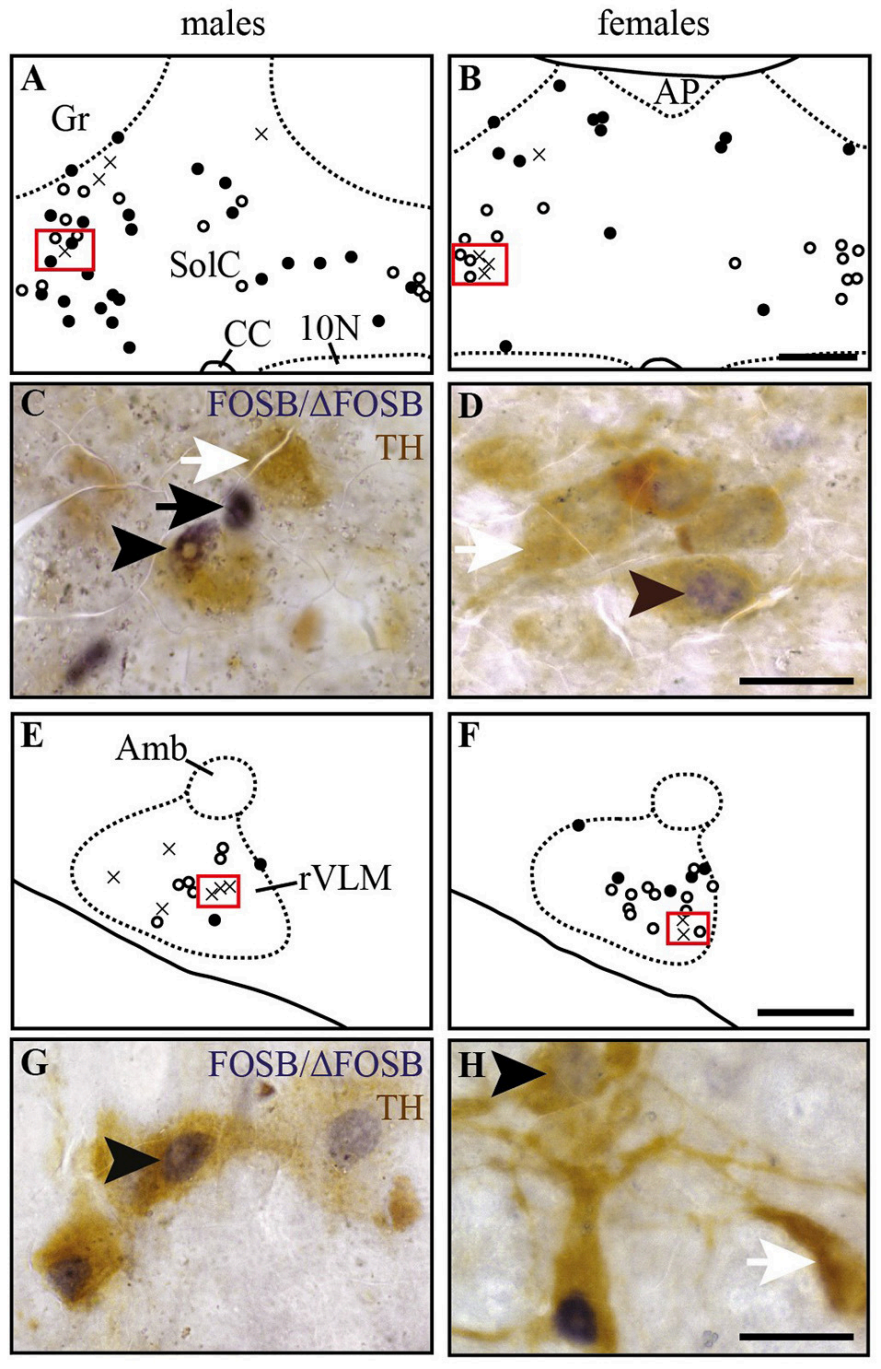
Some of the CIH-induced FOSB/ΔFOSB-positive cells in the Sol and VLM are catecholaminergic. Drawings illustrating the distribution of cells immunoreactive for FOSB/ΔFOSB (black dots), TH (white dots), or both (crosses) under CIH in the SolC **(A,B)** and rVLM **(E,F)** in male **(A,E)** and female **(B,F)** mice. The photomicrographs below the drawings correspond to the regions outlined by the red rectangles of **A,B,E,F** in the SolC **(C,D)** and rVLM **(G,H)** in male **(C,G)** and female **(D,H)** mice. Black and white arrows indicate FOSB/ΔFOSB- and TH-positive cells, respectively, and arrow heads indicate cells immunoreactive for both FOSB/ΔFOSB and TH. Scale bars = 200 μm **(A,B,E,F)** and 20 μm **(C,D,G,H)**. 10N, dorsal motor nucleus of vagus; Amb, ambiguus nucleus; CC, central canal; Gr, gracile nucleus; rVLM, rostral part of the ventrolateral reticular nucleus of the medulla; SolC, nucleus of the solitary tract, commissural part.

**Figure 5:**
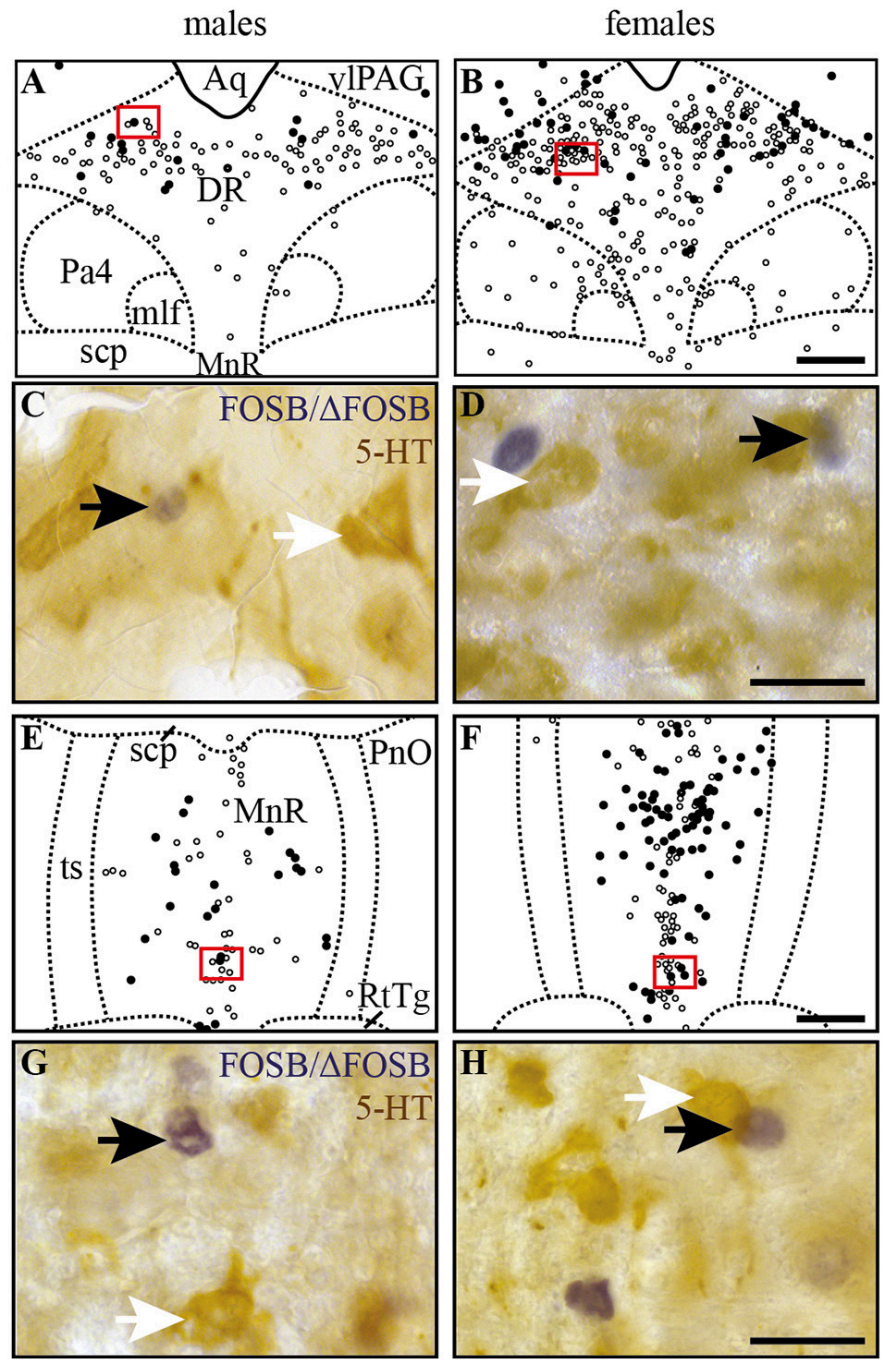
CIH-induced FOSB/ΔFOSB-positive cells in raphe nuclei do not immunolabel for serotonin. Drawings illustrating the distribution of cells immunoreactive for FOSB/ΔFOSB (black dots) or 5-HT (white dots) under CIH in the DR **(A,B)** and MnR **(E,F)** in male **(A,E)** and female **(B,F)** mice. The photomicrographs below the drawings correspond to the regions outlined by the red rectangles of **A,B,E,F** in the DR **(C,D)** and MnR **(G,H)** in male **(C,G)** and female **(D,H)** mice. Black and white arrows indicate FOSB/ΔFOSB- and 5-HT-positive cells, respectively. Scale bars = 200 μm **(A,B,E,F)** and 20 μm **(C,D,G,H)**. Aq, aqueduct; DR, dorsal raphe nucleus; mlf, medial longitudinal fasciculus; MnR, median raphe nucleus; Pa4, paratrochlear nucleus; PnO, pontine reticular nucleus, oral part; RtTg, reticulotegmental nucleus of the pons; scp, superior cerebellar peduncle; ts, tectospinal tract; VLPAG, ventrolateral periaqueductal gray.

**Figure 6:**
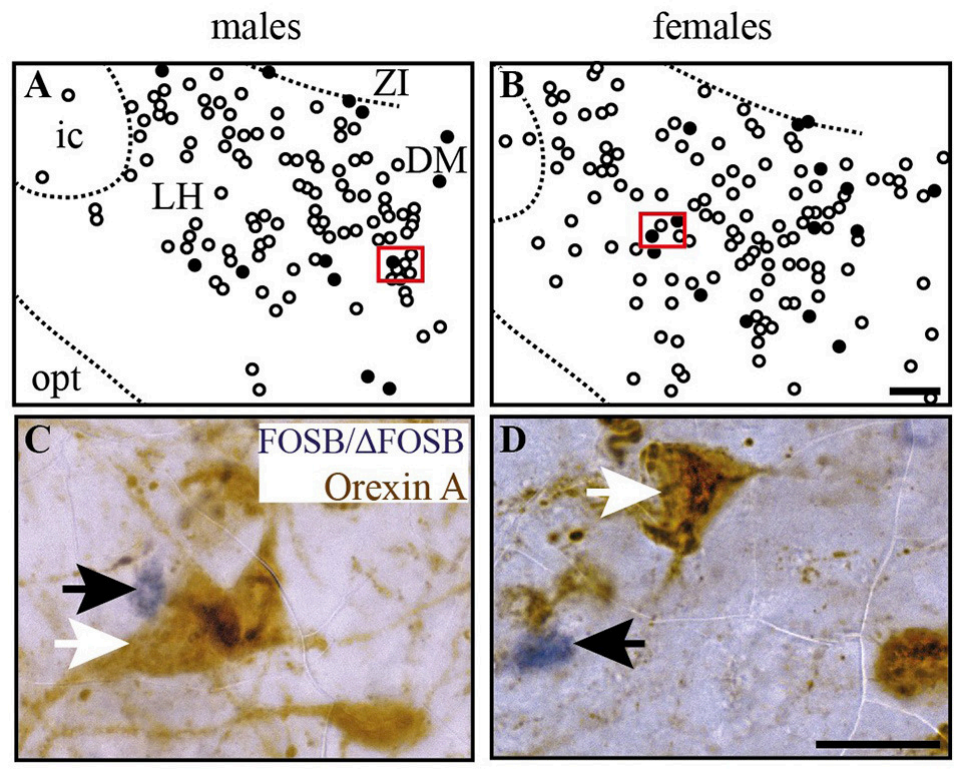
CIH-induced FOSB/ΔFOSB-positive cells in the caudal hypothalamus are not immunolabeled for orexin. Drawings illustrating the distribution of cells immunoreactive for FOSB/ΔFOSB (black dots) and orexin (white dots) under CIH in the caudal hypothalamus **(A,B)** in male **(A)** and female **(B)** mice. The photomicrographs below the drawings correspond to the regions outlined by the red rectangles **(A,B)** in male **(C)** and female **(D)** mice. Scale bars = 100 μm **(A,B)** and 20 μm **(C,D)**. DM, dorsomedial hypothalamic nucleus; ic, internal capsule; LH, lateral hypothalamic area; opt, optic tract; Zi, zona incerta.

Sections were examined under a light microscope (DM-200-LED; Leica Microsystems, Heidelberg, Germany). FOSB/ΔFOSB-positive cells were analyzed in brainstem and diencephalic structures related to cardiorespiratory control (Figures 1–6): hypoglossal nucleus (12N), A5 region (A5), dorsolateral periaqueductal gray (DLPAG), dorsomedial periaqueductal gray (DMPAG), dorsomedial hypothalamic nucleus (DM), dorsal raphe nucleus (DR), locus coeruleus (LC), lateral hypothalamic area (LH), lateral parabrachial nucleus (lPB), lateral paragigantocellular nucleus (LPGi), median raphe nucleus (MnR), medial parabrachial nucleus (mPB), posterior hypothalamic area (PH), raphe magnus nucleus (RMg), raphe obscurus nucleus (ROb), raphe pallidus nucleus (RPa), nucleus of the solitary tract, commissural part (SolC), median part (SolM), and ventrolateral part (SolVL), subcoeruleus nucleus (SubC), VLM, ventrolateral reticular nucleus of the medulla caudal part (cVLM) and rostral part (rVLM), and ventrolateral periaqueductal gray (VLPAG). The definitions of boundaries of these structures were made according to a mouse brain atlas (Paxinos and Franklin, 2001) with the aid of numerous ventral, dorsal, and lateral landmarks (such as those indicated in Figures 1, 3–6). The VLM is a neuronal column ventral to the *ambiguus* nucleus that includes the A1C1 group of neurons and extends from the pyramidal decussation to the caudal edge of the facial nucleus (Voituron et al., 2006, 2011; Huckstepp et al., 2015). We made a distinction between the caudal part of the VLM (cVLM; from the pyramidal decussation to the caudal edge of the lateral paragigantocellulaire nucleus) and the rostral part of the VLM (rVLM; from the caudal edge of the lateral paragigantocellulaire nucleus to the caudal edge of the facial nucleus) using standard landmarks, as previously described (Voituron et al., 2011; Joubert et al., 2016).

Counts for FOSB/ΔFOSB-positive cells were performed by eye at x400. For dual labeling, the counts were performed by eye at x400 except for the LC, RPa, Rob, and DR where x1000 magnification was used due to the high density of TH and 5-HT labeled cells. In addition, the distribution of FOSB/ΔFOSB-positive cells was plotted onto drawings in order to illustrate their distribution (Figures 1, 3–6), and FOSB/ΔFOSB and double-labeled cells were photographed with a digital camera (Leica DFC450C, Leica Microsystems, Heidelberg, Germany; Figures 4–6). Bilateral structures were analyzed on both left and right sides and the obtained values pooled. For single-labeled cells, the results are expressed as the mean ± SD number of FOSB/ΔFOSB-positive cells per encephalic structure (Table 1). For double-labeled cells, the results are expressed as the mean ± SD percentage of double-labeled cells among the total number of FOSB/ΔFOSB cells in each defined structure.

**Table 1:** Average number of FOSB/ΔFOSB-positive cells throughout brainstem and diencephalic cardiorespiratory structures under baseline and CIH conditions.

	**Sham**		**CIH**	
	**Males**	**Females**	**Males**	**Females**
	6 ≤ *n* ≤ 11	10 ≤ *n* ≤ 11	7 ≤ *n* ≤ 12	8 ≤ *n* ≤ 11
**MEDULLA OBLONGATA**				
12N	10.3 ± 6.3	33.2 ± 19.2^##^	25.0 ± 17.3	28.8 ± 10.8
cVLM	60.7 ± 17.9	70.1 ± 35.1	75.6 ± 46.4	94.2 ± 47.8
rVLM	20.5 ± 20.2	38.4 ± 19.5^#^	63.7 ± 18.9^***^	60.3 ± 13.4
SolC	24.2 ± 16.4	19.7 ± 10.8	61.9 ± 22.5*	45.1 ± 25.9¤
SolM	139.3 ± 31.0	135.2 ± 36.5	210.0 ± 87.2	159.9 ± 73.5
SolVL	68.0 ± 14.0	64.6 ± 22.0	92.3 ± 28.3	64.9 ± 26.5
ROb	1.8 ± 2.6	2.9 ± 4.6	0.4 ± 0.8	1.9 ± 1.6
RPa	2.3 ± 3.8	3.9 ± 6.3	1.9 ± 2.3	5.1 ± 4.5
LPGi	27.0 ± 11.1	23.6 ± 4.9	28.4 ± 4.1	27.8 ± 8.1
**PONS**				
A5	6.8 ± 5.9	6.4 ± 3.0	14.0 ± 5.5	20.7 ± 11.4^¤¤^
LC	45.7 ± 39.3	44.5 ± 19.3	50.6 ± 17.1	48.3 ± 22.1
SubC	61.0 ± 38.1	73.0 ± 22.9	83.1 ± 23.1	92.8 ± 35.2
lPB	159.3 ± 42.8	195.0 ± 94.7	157.1 ± 56.8	242.3 ± 66.4
mPB	103.3 ± 47.6	93.4 ± 33.4	78.9 ± 43.0	118.4 ± 35.3
RMg	39.2 ± 13.4	26.3 ± 6.5	46.9 ± 18.3	25.9 ± 8.6^§^
**MESENCEPHALON**				
DLPAG	299.5 ± 94.9	365.0 ± 99.2	313.3 ± 100.2	481.5 ± 115.3^§^
DMPAG	42.7 ± 17.5	48.6 ± 17.0	55.1 ± 17.2	103.6 ± 37.0^§^,^¤¤^
VLPAG	215.0 ± 58.3	228.7 ± 34.7	218.4 ± 74.3	280.1 ± 98.2
DR	55.8 ± 16.8	140.6 ± 49.1^####^	128.0 ± 37.4^***^	177.8 ± 52.7
MnR	33.7 ± 28.3	120.6 ± 41.9^####^	66.6 ± 37.5*	156.3 ± 50.4^§^
**DIENCEPHALON**				
DM	84.2 ± 30.8	186.1 ± 61.2^##^	142.9 ± 50.6	242.7 ± 108.6^§§^
LH	271.7 ± 79.3	335.1 ± 104.0	257.1 ± 109.4	350.6 ± 108.0
PH	138.5 ± 82.5	230.3 ± 90.2	139.1 ± 85.0	350.8 ± 130.7^§§§§^,^¤^

Values presented are total numbers of FOSB/ΔFOSB-positive cells per structure ± S.D.

*, *** sham vs CIH for males, p < 0.05, 0.001 respectively;

¤, ¤¤ sham vs CIH for females, p < 0.05, 0.01, respectively;

#, ##, #### males vs females for sham conditions, p < 0.05, 0.01, 0.0001 respectively;

§, §§, §§§§ *males vs females for CIH conditions, p < 0.05, 0.01, 0.0001, respectively*.

*Please, noted that depending on normality, all analyzed structures except the DM, LH and PH, have been analyzed via a non-parametric test. Number of symbols in increasing order signify p < 0.05, p < 0.01, p < 0.001, p < 0.0001. 12N, hypoglossal nucleus; A5, A5 noradrenaline cells; cVLM, caudal part of the ventrolateral reticular nucleus of the medulla; DLPAG, dorsolateral periaqueductal gray; DM, dorsomedial hypothalamic nucleus; DMPAG, dorsomedial periaqueductal gray; DR, dorsal raphe nucleus; LC, locus coeruleus; LH, lateral hypothalamic area; lPB, lateral parabrachial nucleus; LPGi, lateral paragigantocellular nucleus; MnR, median raphe nucleus; mPB, medial parabrachial nucleus; PH, posterior hypothalamic area RMg, raphe magnus nucleus; ROb, raphe obscurus nucleus; RPa, raphe pallidus nucleus; rVLM, rostral part of the ventrolateral reticular nucleus of the medulla; SolC, nucleus of the solitary tract, commissural part; SolM, nucleus of the solitary tract, medial part; SolVL, nucleus of the solitary tract, ventrolateral part; SubC, subcoeruleus nucleus; VLPAG, ventrolateral periaqueductal gray*.

### Quantitative analysis of the effect of chronic intermittent hypoxia on serotoninergic innervation of the 12N

**Figure 7:**
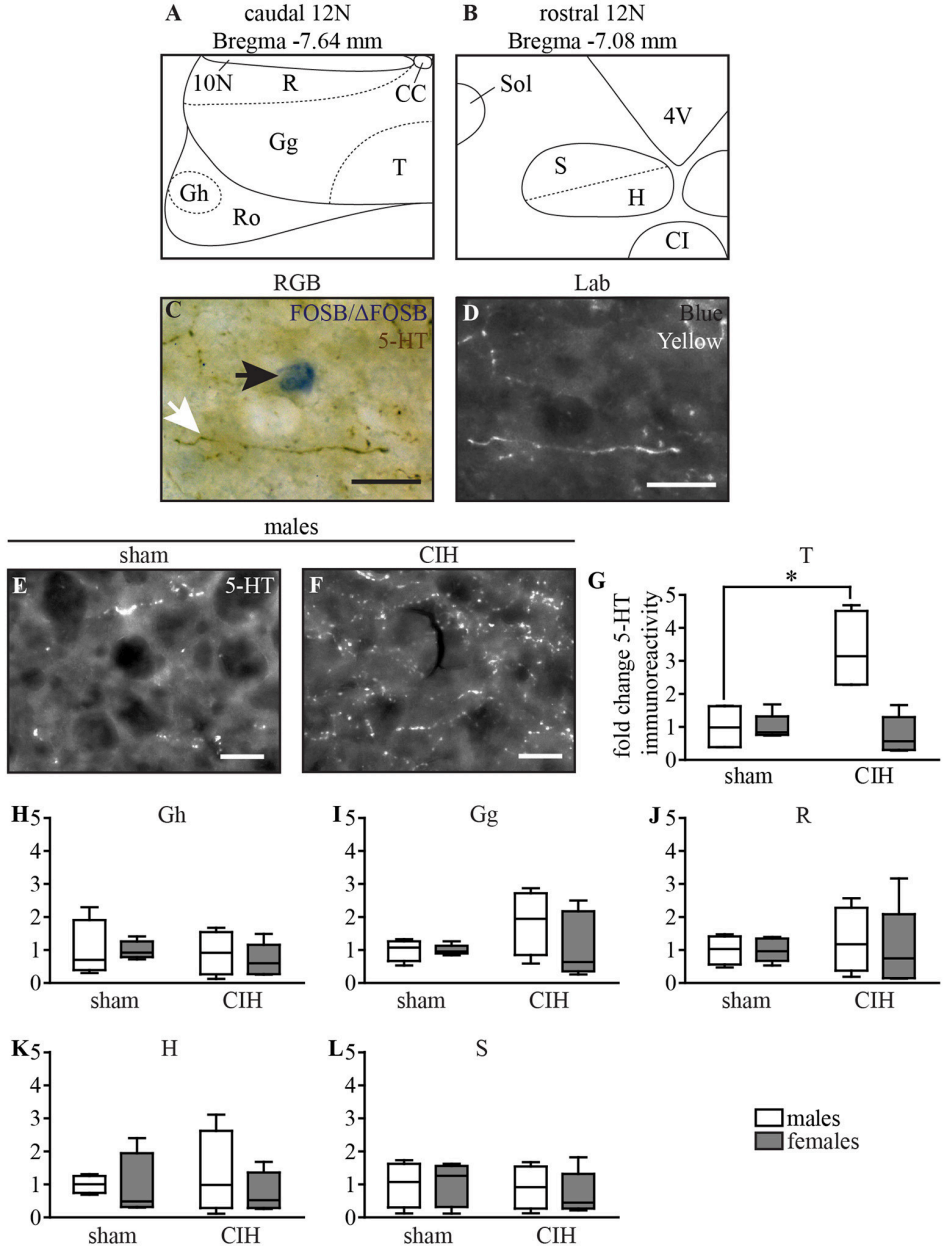
CIH increases 5-HT immunoreactivity in the ventromedial part of the caudal 12N in male but not female mice. Schematic representation of the subdivisions of the 12N, according to its myotonic organization in the caudal **(A)** and rostral **(B)** parts. Photomicrographs of the 12N in RGB (Red, Green, Blue, **C**) illustrating a FOSB/ΔFOSB-positive cell (black arrow) and a 5-HT immunoreactive fiber (white arrow) and converted to L^*^a^*^b (**D**, shown is in the “b” channel which segregates the blue and yellow stains). Images from the b channel of the L^*^a^*^b format in the T subdivision of the 12N under sham conditions **(E)** and CIH **(F)** in male mice. Scale bars = 20 μm. Box plot showing changes in 5-HT immunoreactivity in the 12N subdivisions for male and female mice between sham and CIH conditions in the T (**G**, intrinsic tongue), Gh (**H**, geniohyoid), Gg (**I**, genioglossus), R (**J**, retrusor), H (**K**, hyoglossus), and S (**L**, styloglossus) subdivisions; median, quartiles, minima, and maxima. ^*^p < 0.05.

The 5-HT innervation of all subdivisions of the 12N was evaluated using ImageJ 1.51n imaging software on high magnification images (x200, DM-200-LED; Leica Microsystems, Heidelberg, Germany) at bregma −7.64 and −7.08 mm (Figures 7A,B) (Fay and Norgren, 1997; Paxinos and Franklin, 2001). Three images at different focal levels were captured for each selected brain section and then stacked to from a single sharp image of all 5-HT immunoreactivity inside the section. Images were converted to the L^*^a^*^b^*^ format, b channel (blue-yellow color separation), which provided the best possible color separation between FOSB/ΔFOSB and 5-HT stains by attributing a white color to the DAB-stained 5-HT immunoreactivity (Figures 7C,D). We quantified the proportion of the area occupied by 5-HT immunoreactivity within the total area of the 12N, according to its myotopic organization previously identified in rat: in the genioglossus (Gg), geniohyoid (Gh), hyoglossus (H), retrusor (R), styloglossus (S), and intrinsic tongue muscle (T) subdivisions (Krammer et al., 1979; Altschuler et al., 1994; Fay and Norgren, 1997; Schwarz et al., 2009). The T subdivision innervates both intrinsic tongue muscles and the genioglossus muscle (Krammer et al., 1979; Altschuler et al., 1994; Schwarz et al., 2009). The mean between the right and left lateral side was calculated for each subdivision of the 12N and results are expressed as the fold changes ± SD relative to sham conditions for their corresponding sex.

### Analysis of the number of serotoninergic neurons in the DR

The number of 5-HT-immunoreactive neurons in the DR was counted using ImageJ 1.51n imaging software in two fields per animal from high magnitude images [x200, DM-200-LED; Leica Microsystems, Heidelberg, Germany; 1.24 ± 0.03 mm^2^; at bregma 4.6 and −4.8 mm, according to the mouse brain atlas, (Paxinos and Franklin, 2001)]. The impact of CIH on male and female mice was determined by expressing the number of 5-HT neurons under CIH as the fold change relative to the corresponding sham conditions.

### Statistics

We compared the mean values obtained under sham and CIH conditions for males and females and between sexes using GraphPad (GraphPad Prism 5, San Diego California USA). Two-way ANOVA followed by Tukey's multiple comparisons test or non-parametric equivalents were used depending on the normality of the data (D'Agostino & Pearson omnibus normality test). The percentages of dual-labeled cells, the number of 5-HT immunoreactive neurons of the DR, and the amount of 5-HT immunoreactive innervation of the 12N were processed in the same statistical manner. Differences were considered significant for *p* < 0.05.

## Results

### Baseline *Fosb* expression throughout the brainstem and hypothalamus in male and female mice

The baseline numbers of FOSB/ΔFOSB-positive cells observed under sham conditions did not significantly vary in most of the analyzed brainstem and hypothalamic structures between males and females (Table 1). However, females displayed a significantly higher number of FOSB/ΔFOSB-positive cells than males in the 12N, especially in the rostral part (+221%, *p* < 0.01; Table 1, Figures 1E,F), rVLM (+87%, *p* < 0.05; Table 1, Figures 1E,F,I,J), DR (+152%, *p* < 0.0001; Table 1, Figures 1M,N,Q,R), MnR (+258%, *p* < 0.0001; Table 1, Figures 1Q,R), and DM (+121%, *p* < 0.01; Table 1, Figures 3A,B).

### CIH-induced *Fosb* expression in male mice

At the ponto-medullary level, males displayed a significantly higher number of FOSB/ΔFOSB-positive cells following CIH than under sham conditions in the well-known cardiorespiratory structures SolC (+156%; *p* < 0.05; Table 1, Figures 1A,C) and rVLM (+211%; *p* < 0.001; Table 1, Figures 1E,G), but not the SolM (Table 1, Figures 1A,C) or cVLM (Table 1, Figures 1A,C). The proportion of FOSB/ΔFOSB-positive cells co-labeled for TH in these catecholaminergic structures was relatively small, but was nevertheless significantly higher than under sham conditions in the rVLM (12.2 ± 6.4% vs. 1.0 ± 0.5%; *p* < 0.0001; Figures 5E–H) and cVLM (6.5 ± 6.9 vs. 0.8 ± 1.2; *p* < 0.05), but unchanged in the SolC (≈5.2 vs. ≈0.4%) and SolM (≈0.6 vs. ≈0.7%). We also observed a tendency toward a two-fold increase in the number of FOSB/ΔFOSB-positive cells in the 12N (+142%; Table 1, Figures 1E,G) and non-catecholaminergic cells in A5, another well-known cardiorespiratory structure (+105%; Table 1, Figures 1M,O), although the differences were not significant. No changes were observed in other cardiorespiratory ponto-medullary structures, i.e., RPa, ROb, LPGi, lPB, mPB, LC, and SubC (Table 1, Figure 1). The FOSB/ΔFOSB-positive cells in the RPa and ROb were not immunoreactive for 5-HT (data not shown).

At the mesencephalic level, we observed a significantly higher number of FOSB/ΔFOSB-positive cells under CIH than sham conditions in the DR (+129%, *p* < 0.001; Table 1, Figures 1M,O,Q,S) and MnR (+98%, *p* < 0.05; Table 1, Figures 1Q,S). The proportion of FOSB/ΔFOSB-positive cells also immunoreative for 5-HT was virtually null under both CIH and sham conditions (≈0.5% vs. no co-labeled cells and ≈0.3 vs. ≈0.3% for the DR and MnR, respectively; Figure 4). No changes were observed in the PAG, regardless of subdivision (Table 1, Figure 1).

At the diencephalic level, we did not observe any significant changes induced by CIH in the analyzed structures (Table 1, Figures 3A,D,E,G). In addition, the FOSB/ΔFOSB-positive cells were not immunoreactive for orexin (≈0.6 vs. ≈0.4%, CIH and sham conditions, respectively).

### CIH-induced *Fosb* expression in female mice

At the ponto-medullary level, females displayed a significant accumulation of FOSB/ΔFOSB-positive cells under CIH relative to sham conditions in the SolC (+129%, *p* < 0.05; Table 1, Figures 1B,D) and A5 (+225%, *p* < 0.01; Table 1, Figures 1N,P), but not the SolM (Table 1, Figures 1F,H), cVLM (Table 1, Figures 1B,D), or rVLM (Table 1, Figures 1J,L). The observed elevated *Fosb* expression in the SolC and A5 under CIH relative to sham conditions occurred specifically in females in metestrus/diestrus phases (+221%, *p* < 0.01 for SolC; +265%, *p* < 0.05 for A5; Figure 2), but not proestrus/estrus phases. As for males, the proportion of FOSB/ΔFOSB-positive cells co-labeled for TH was higher than under sham conditions in the rVLM (7.7 ± 1.6% vs. 1.0 ± 0.6, *p* < 0.001; Figures 5B,D,F,H), but unchanged in the SolC (≈11.2% vs. ≈8.0%), SolM (≈0.3% *vs* ≈0.4%), cVLM (≈2.0 vs. ≈0.6%), and A5 (≈1.0% vs. ≈2.1%). No changes were observed in the other analyzed ponto-medullary structures, i.e., 12N, RPa, ROb, LPGi, lPB, mPB, LC, and SubC (Table 1, Figure 1). Also, as for males, the FOSB/ΔFOSB-positive cells in the RPa and ROb were not immunoreactive for 5-HT (data not shown). The number of FOSB/ΔFOSB-positive cells of the RMg under CIH was significantly lower in females than males (−45%, *p* < 0.05; Table 1, Figures 1K,L).

At the mesencephalic level, in contrast to males, females did not display any increase in FOSB/ΔFOSB-positive cells in the DR (Table 1, Figures 1N,P,S,T) or MnR (Table 1, Figures 1S,T), but displayed a significant increase in FOSB/ΔFOSB-positive cells in the DMPAG (+113%, *p* < 0.01; Table 1, Figures 1S,T). In addition, the number of FOSB/ΔFOSB-positive cells under CIH was significantly higher in females than males in the DLPAG (+88%, *p* < 0.05; Table 1, Figures 1S,T), DMPAG (+54%, *p* < 0.01; Table 1, Figures 1S,T), and MnR (+135%, *p* < 0.01; Table 1, Figures 1S,T).

At the diencephalic level, female mice displayed a significant increase in FOSB/ΔFOSB-positive cells in the PH (+152%, *p* < 0.05; Table 1, Figures 3B,D) but not the DM (Table 1, Figures 3B,D) or LH (Table 1, Figures 3B,D). As for males, the FOSB/ΔFOSB-positive cells in the caudal hypothalamus were not immunoreactive for orexin (≈0.3% vs. ≈0.1%, CIH and sham conditions, respectively). In addition, the number of FOSB/ΔFOSB-positive cells was significantly higher in females than males in the DM (+70%, *p* < 0.01; Table 1, Figures 1C,D) and PH (+152%, *p* < 0.001; Table 1, Figures 1C,D).

### CIH-induced changes in the serotoninergic innervation of the 12N in male but not female mice

5-HT-immunoreactivity in the T subdivision of the 12N, innervating intrinsic tongue and genioglossus muscles (Krammer et al., 1979; Fay and Norgren, 1997), increased in males upon CIH stimulation relative to sham conditions (+231%, *p* < 0.05; Figures 7E–G). We did not observe such an effect in other subdivisions of the 12N in males (Figures 7H–L), although there was a tendency in the Gg subdivision (Figure 7I) and in all 12N subdivisions in females (Figures 7G–L).

### CIH-induced loss of 5-HT immunoreactive neurons in the DR in male but not in female mice

**Figure 8:**
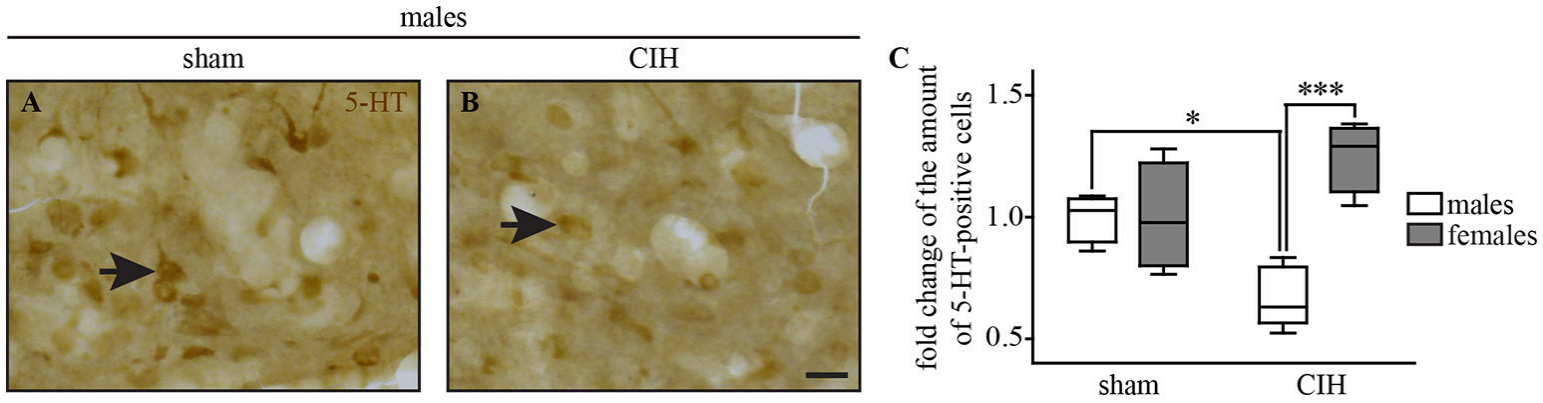
The number of 5-HT-positive cells in the dorsal raphe nucleus decreases under CIH in male but not female mice. Photomicrographs of the DR nucleus in male mice under sham **(A)** and CIH conditions **(B)**. Box plot showing the number of 5-HT immunoreactive neurons in male and female mice between sham and CIH conditions in the DR; median, quartiles, minima, and maxima **(C)**. Scale bar = 20 μm. ^*^p < 0.05; ^***^p < 0.001.

We observed a significant decrease in the number of 5-HT-positive cells in the DR in male but not female mice under CIH relative to sham conditions (−32.9%; *p* < 0.05; Figure 8), whereas the baseline number of 5-HT-positive neurons was similar between males and females under sham conditions.

## Discussion

We highlighted sexual dimorphisms in the neuroplasticity induced by CIH in mice by an extensive analysis of the distribution of FOSB/ΔFOSB-positive neurons in cardiorespiratory structures. Our main finding is that female mice displayed higher baseline and CIH-induced *Fosb* expression than male mice, suggesting that females exhibit greater neuroplastic dynamics, resulting in better compensation to CIH stimulation than males. In particular, our results revealed sex-based differences in CIH-induced neuroplasticity at the level of the sympathoexcitatory rVLM between males and females, suggesting that this structure could be a key element for the sex-based difference in hypertension already reported (Hinojosa-Laborde and Mifflin, 2005; Huang et al., 2008; Yu et al., 2014). We also observed sexual dimorphism in serotoninergic systems induced by CIH that may contribute to the decrease in upper airway stability in males (O'Donnell et al., 2000; Jordan et al., 2004; Chin et al., 2012).

### Sex-based differences in baseline and CIH-induced *Fosb* expression suggest that female mice display higher neuroplastic capacities than males

Our primary observation was that the number of baseline and CIH-induced FOSB/ΔFOSB-positive neurons were more numerous in female than male mice in many cardiorespiratory structures, i.e., 12N, rVLM, DLPAG, DMPAG, DR, MnR, DM, and PH. High numbers of FOSB/ΔFOSB-positive neurons in females could result from estrogen signaling, which is more potent in females and shown to induce CREB-mediated *c-Fos* expression (Duan et al., 1998, 2001) and possibly expression of *Fosb*, since the promoter regions are conserved between both genes (Lazo et al., 1992; Herdegen and Leah, 1998). Consistent with this hypothesis, neurons in the 12N, DR, and hypothalamic region, containing the DM and PH, have been shown to express higher levels of estrogen receptor alpha (ERα) in females than males (Lauber et al., 1991; Vanderhorst et al., 2005; Schlenker and Hansen, 2006). In contrast, there is no data in the literature suggesting such an elevation in females in the PAG and MnR. Given the characteristics of *Fosb*, the higher number of FOSB/ΔFOSB-positive neurons in cardiorespiratory structures in females than males suggests that females have a high number of neurons with high transcriptional potential in these structures. Indeed, FOSB and its splice variant ΔFOSB, both products of *Fosb* expression, are transcriptional co-factors that heterodimerize with JUN family proteins to form active activator protein-1 (AP-1), which binds to AP-1 sites in the promoter regions of target genes and regulates their expression (Herdegen and Leah, 1998; Nestler, 2008). Thus, FOSB/ΔFOSB has been shown to modulate gene expression, promoting, for example, decreases in the conductance of the alpha-amino-3-hydroxy-5-methyl-4-isoxazolepropionic acid (AMPA) glutamate receptor (Kelz et al., 1999) or changes in the phosphorylation state of numerous synaptic proteins by increasing cyclin-dependent kinase-5 gene expression (Bibb et al., 2001). The high number of FOSB/ΔFOSB-positive neurons in females may thus reflect greater neuroplastic dynamics than males and we hypothesize that this high remodeling capacity in females provides advantages in maintaining cardiorespiratory homeostasis. This hypothesis is supported by several published studies. First, it has been shown that neuroplasticity by synaptic remodeling is a process that partially depends on estrogen signaling (Srivastava et al., 2013) and second, *Fosb* knock-out mice lack any form of hypoxia-induced respiratory plasticity (Malik et al., 2005). In addition, it has been shown that neuroplastic processes are crucial for maintaining respiratory homeostasis (Streeter and Baker-Herman, 2014; Braegelmann et al., 2017). This hypothesis is also consistent with the previously reported higher susceptibility of males than females to perturbations in genes involved in synaptic plasticity (Mottron et al., 2015). Thus, our present observations may provide new insights into the protective mechanisms of estrogen found in CIH in addition to its antioxidant activity (Borrás et al., 2003; Sanfilippo-Cohn et al., 2006; Zhang et al., 2009). Thus, as discussed in the following paragraph, weaker CIH-induced cardiorespiratory alterations, such as hypertension (Hinojosa-Laborde and Mifflin, 2005) and hyperventilation in female rats relative to males, may be associated with differential CIH-induced neuroplasticity because of a central effect of estrogen, as previously suggested (Zabka et al., 2001a,b; Skelly et al., 2012). Measuring the level of circulating estrogens in females in the future could provide even greater insights into the influence of female sex hormones, such as estrogen, on *Fosb* expression dynamics and cardiorespiratory homeostasis. Of course, such protective mechanisms may also depend on female steroid sex hormones other than estrogen. In addition, it is noteworthy that the distinction between metestrus/diestrus and proestrus/estrus in female mice showed a difference in CIH-induced *Fosb* expression in a few structures i.e., the SolC and A5, supporting a link between sex-dependent neuroplasticity under CIH and female sex hormones.

### Similarities and differences in CIH-induced neuroplasticity in brainstem sympathoexcitatory structures

Male mice displayed an elevation in the number of FOSB/ΔFOSB-positive cells in brainstem structures previously reported to be involved in CIH-induced hypertension in male rats, i.e., the SolC and the rVLM (Knight et al., 2011; Cunningham et al., 2012; Faulk et al., 2017). Although increased *Fosb* expression in the SolC has been associated with the hypertension developed during CIH exposure (Cunningham et al., 2012), in the rVLM, it has been associated with sustained hypertension observed during the normoxic period that follows CIH exposure (Cunningham et al., 2012; Faulk et al., 2017). Thus, it is very likely that the CIH-induced neuroplasticity observed in the SolC and rVLM of male mice is associated with the development of hypertension, as already reported in male mice (Schulz et al., 2014). The SolC is the primary target of peripheral afferent projections from the carotid body, considered to be the main dioxygen chemoreceptor organ (Finley and Katz, 1992; Iturriaga and Alcayaga, 2004). CIH increases both the basal discharges of the carotid bodies in normoxia and their response to acute hypoxia (Iturriaga et al., 2009). In addition, coupling of the carotid body/SolC is considered to strongly influence the development of CIH-induced hypertension because denervation of the bilateral carotid bodies before CIH exposure prevents the development of hypertension in rats (Fletcher et al., 1992a).

In recent years, many observations have shown that the production of reactive oxygen species (ROS) during CIH is an essential mechanism of hypoxia-mediated elevation of carotid-body activity and the cardiovascular consequences (Rey et al., 2004; Garvey et al., 2009; Iturriaga et al., 2009; Del Rio et al., 2010, 2011). ROS production is reduced by estrogens, which are considered to be potent antioxidants (Moorthy et al., 2005a). We thus expected to find fewer neurons in the SolC expressing *Fosb* in female than male mice under CIH because of potentially less stimulation by the carotid body in females. Surprisingly, we observed a greater number of FOSB/ΔFOSB-positive neurons in the SolC of females than males. However, the CIH-induced increase in the number of FOSB/ΔFOSB-positive neurons in the SolC occurred during metestrus/diestrus phases, but not proestrus/estrus phases. We hypothesized that it is prevented by high levels of female sex hormones. Thus, the estrogen-mediated reduction of ROS production in females appears to be sufficient to significantly decrease the elevation of the activity of the carotid bodies by CIH and thus decrease the stimulation of SolC neurons. This would suggest that the lower prevalence of hypertension in women than men OSA patients (Huang et al., 2008; Yu et al., 2014) and the reported reduced severity of hypertension developed by female than male rats (Hinojosa-Laborde and Mifflin, 2005) are likely to result from sexual dimorphism in the SolC. In addition, female mice did not display a significant increase in the number of FOSB/ΔFOSB-positive cells in the rVLM, in contrast to male mice, even if the proportion of dually labeled cells for FOSB/ΔFOSB and TH was higher in females than males under CIH. The rVLM is a key sympathoexcitatory blood pressure center that contains neurons projecting to preganglionic neurons (Brown and Guyenet, 1984; Guyenet, 2006). Activity of these sympathoexcitatory neurons is under the direct or indirect control of several brain structures, including the SolC (Guyenet, 2006). It is possible that there is a differential effect of CIH on one or more neurotransmission systems that regulate the activity of the rVLM sympathoexcitatory neurons. It may be informative to search for sex-dependent differences in protein expression in rVLM between male and female mice exposed to CIH, as already done for the vascular wall (Li et al., 2014). Further studies are necessary, first to determine the origin of the difference in the effect of CIH on rVLM neurons between females and males and second, to determine whether this difference in CIH-induced neuroplasticity is followed by less hypertension in female than male mice. Recent work of Laouafa et al. shows that estradiol prevents the cardiorespiratory disorders induced by CIH (Laouafa et al., 2017). It is thus also possible that the reduction of ROS production induced by estradiol (Moorthy et al., 2005b; Arevalo et al., 2015) participates in reducing the activation of rVLM neurons and thus contributes to limit the cardiovascular consequences of CIH in females.

### CIH increases 5-HT immunoreactivity in the hypoglossal nucleus that controls tone of the upper airway muscles in males but not in females

CIH induced an increase in 5-HT immunoreactivity in the T subdivision of the 12N in male but not female mice. Such an increase has been reported in male rats (Rukhadze et al., 2010). The T subdivision contains motoneurons that innervate intrinsic tongue muscles and the genioglossus (Krammer et al., 1979; Altschuler et al., 1994; Schwarz et al., 2009), both involved in opening of the upper airways (Fregosi and Ludlow, 2014). Data in the literature describe the involvement of 5-HT in maintaining appropriate tone of upper airway muscles in animals (Veasey et al., 1996, 1999, 2001; Carley and Radulovacki, 1999; Nakano et al., 2001; Ogasa et al., 2004; Fenik et al., 2005; Zhong et al., 2010). Additionally, patients suffering from depression and characterized by 5-HT-system deficiency have a high prevalence of OSA (Saunamäki and Jehkonen, 2007; Hein et al., 2017). In this context, the observed neuroplasticity of 5-HT systems near hypoglossal motoneurons in male mice may suggest that the repetitive episodes of hypoxia encountered by OSA patients may affect the tone of the upper airway muscles in men, but not women. This is supported by various data from the literature. First, it has been described in male rodents that CIH is sufficient to induce upper airway muscle dysfunction (Ray et al., 2007; Conotte et al., 2016). Second, clinical observations have concluded that the tone of the upper airway muscles is lower in male than female OSA patients (O'Donnell et al., 2000; Jordan et al., 2004; Chin et al., 2012). Third, genetic polymorphisms of the 5-HT systems are associated with OSA in men, but not women (Ylmaz et al., 2005). Mechanisms by which sex hormones participate in the difference between men and women concerning upper airway collapsibility are not fully understood, even if it has been recently reported in mice that estrogens play a key role, partially by antioxidant activity, within upper airway muscles (O'Halloran et al., 2017).

Thus, in this context, we hypothesize that the observed increase in 5-HT immunoreactivity in the T subdivision of the 12N of male mice is associated with a decrease in 5-HT release that would result from an accumulation of 5-HT in neuronal terminations. Such a phenomenon has already been proposed in another context (Curtis et al., 2013). Although a 5-HT immunolabeling alone does not allow conclusive determination of whether 5-HT release was affected, the previously described decrease in 5-HT release in 12N under CIH reinforces this hypothesis (Wu et al., 2017). Such a decrease is compatible with observations from clinical and animal studies, leading to the proposition of 5-HT drugs as a potential therapy for OSA (Veasey et al., 1996, 1999, 2001; Carley and Radulovacki, 1999; Nakano et al., 2001; Ogasa et al., 2004; Saunamäki and Jehkonen, 2007; Prasad et al., 2010; Zhong et al., 2010; Hein et al., 2017). This may thus contribute to the lower upper airway muscle tone described in male subjects and male mice than that of females (O'Donnell et al., 2000; Jordan et al., 2004; Chin et al., 2012; O'Halloran et al., 2017), because of a decrease in the excitatory influence of 5-HT on hypoglossal motoneurons (Fenik et al., 1997, 2005; Prasad et al., 2010). Combining data from the literature and our FOSB/ΔFOSB results, we propose that the increase in 5-HT immunoreactivity in the 12N of males involves a decrease in the activity of 5-HT neurons of the DR. The DR is considered to be the major serotonin containing nucleus of the brainstem (Steinbusch and Nieuwenhuys, 1983) and is one of the raphe nuclei known to contain neurons directly projecting to the 12N (Vertes and Kocsis, 1994). Two mechanisms are likely to be involved in alterations of the transmission of 5-HT from the DR to the 12N. First, the CIH-induced partial loss of 5-HT-immunoreactive neurons in the male DR could lead to decreased release of 5-HT in the 12N, which is consistent with findings of another study (Wu et al., 2017). This probably results from oxidative stress, which particularly affects male rodents in CIH (Borrás et al., 2003; Sanfilippo-Cohn et al., 2006; Deng et al., 2015). Second, the DR-mediated transmission of 5-HT to the 12N could be actively counteracted by non-serotoninergic cell populations of the DR. In our study, we observed an increase in FOSB/ΔFOSB-positive cells in the DR induced by CIH in males but not females. We suggest that these FOSB/ΔFOSB-positive cells are adjacent GABAergic interneurons, known to inhibit DR 5-HT neurons (Levine and Jacobs, 1992; Fenik et al., 1997, 2005), because they were not immunolabeled for 5-HT. Consistent with this hypothesis, GABA-mediated 5-HT inhibition may occur in a sex-dependent manner and be stronger in males (Felton and Auerbach, 2004; Dergacheva, 2015). Thus, the CIH-induced increase in activity of non-5-HT neurons of the DR, suggested by *Fosb* expression, and partial 5-HT cell loss in males could both be key processes in the reported greater upper airway collapsibility of men than women (O'Donnell et al., 2000; Jordan et al., 2004; Chin et al., 2012). As previously mentioned, the absence of a change in the number of FOSB/ΔFOSB-positive cells in the DR in females under CIH may be related with their greater neuroplasticity dynamics and thus their greater ability to more efficiently adapt to environmental changes than males. Further studies are necessary to better understand the mechanisms involved in this protective effect in females, but it is very likely that estrogens participate, at least in part, as they participate in the resilience to CIH-induced dysfunction of upper airway muscles in females (O'Halloran et al., 2017).

## Conclusion

This study significantly contributes to the knowledge of similarities and differences in the CIH-induced neuroplasticity between males and females, making it possible to highlight potential mechanisms behind the functional differences observed between women and men with OSA (O'Donnell et al., 2000; Jordan et al., 2004; Huang et al., 2008; Chin et al., 2012; Yu et al., 2014) and male and female rodents subjected to CIH (Hinojosa-Laborde and Mifflin, 2005; Skelly et al., 2012). Our results show that females display overall greater neuroplastic potential than males, suggesting that female sex hormones drive the elevated homeostatic potential in the context of CIH. In particular, our data highlight first, a difference between males and females at the level of the sympathoexcitatory rVLM structure that may participate to limit the development of hypertension in females under CIH and second, differential neuroplasticity of the 5-HT systems in the hypoglossal nucleus, which predisposes males to greater alteration of the neuronal control of the upper respiratory tract. FOSB/ΔFOSB however, was not limited to structures that have been linked to cardiorespiratory comorbidities of OSA, suggesting that CIH impairs several systems that need to be further investigated.

## Author contributions

DB performed experiments, analyzed data, generated the figures and wrote the manuscript. MS performed experiments and analyzed data. FJ participated in obtaining tissues. NV participated in obtaining tissues and made comments on the manuscript. CP made comments on the manuscript. PC designed experiments, discussed the results and their significance and made comments on the manuscript. M-NF, obtained funding, designed experiments, discussed the results and their significance and made comments on the manuscript. LB designed experiments, shaped, and interpreted the data, discussed the results, and their significance, and wrote the manuscript.

### Conflict of interest statement

The authors declare that the research was conducted in the absence of any commercial or financial relationships that could be construed as a potential conflict of interest. The reviewer VJ declared a past co-authorship with one of the authors NV to the handling Editor.

## Abbreviations

12N: hypoglossal nucleus
5-HT: serotonin
7n: facial nerve
7N: facial nucleus
A5: A5 region
Amb: *ambiguus* nucleus
CIH: chronic intermittent hypoxia
CREB: cAMP responsive element binding protein
cVLM: caudal part of the ventrolateral reticular nucleus of the medulla
DLPAG: dorsolateral part of the periaqueductal gray
DM: dorsomedial hypothalamic nucleus
DMPAG: dorsomedial part of the periaqueductal gray
DR: dorsal raphe nucleus
ER: estrogen receptor
KF: Kölliker-Fuse nucleus
LC: locus coeruleus
LH: lateral hypothalamic area
lPB: lateral parabrachial nucleus
mPB: medial parabrachial nucleus
OSA: obstructive sleep apnea
PH: posterior hypothalamic area
RMg: raphe magnus nucleus
ROb: raphe obscurus nucleus
ROS: reactive oxygen species
RPa: raphe pallidus nucleus
SolC: commissural part of the nucleus of the solitary tract
SolM: medial part of the nucleus of the solitary tract
SolVL: ventrolateral part of the nucleus of the solitary tract
rVLM: rostral part of the ventrolateral reticular nucleus of the medulla
SubC: ventral part of the subcoeruleus nucleus
TH: Tyrosine hydroxylase
VLPAG: ventrolateral part of the periaqueductal gray.
